# The impact of yoga on teachers’ self-rated emotions

**DOI:** 10.1186/s13104-019-4737-7

**Published:** 2019-10-22

**Authors:** Shirley Telles, Sachin Kumar Sharma, Ram Kumar Gupta, Deepak Kumar Pal, Kumar Gandharva, Acharya Balkrishna

**Affiliations:** Patanjali Research Foundation, Patanjali Yogpeeth, Haridwar, Uttarakhand 249402 India

**Keywords:** Teachers, Yoga, Mental well-being, State anxiety

## Abstract

**Objectives:**

To assess (i) if teachers’ age or gender could predict their baseline levels of mental well-being and anxiety and any change after yoga. (ii) Whether mental well-being or anxiety changed following 15 days of yoga in primary school teachers. Primary school teachers took part in this single group longitudinal trial (n = 302, group mean age ± SD; 41.8 ± 5.90 years). They received 240 min of yoga practice and 120 min of yoga theory each day. At baseline and after 15 days of yoga the assessments were (i) mental well-being (Warwick-Edinburgh scale) and (ii) state anxiety (Spielberger’s State Trait Anxiety Inventory).

**Results:**

Gender acted as a significant predictor for mental well-being scores (P = 0.001) and state anxiety (P = 0.005) in the group at baseline. Females showed higher anxiety scores and lower mental well-being scores. Following yoga the teachers showed a significant increase in mental well-being by 5.84% and a decrease in state anxiety by 4.48%.

*Trial registration* The trial was registered retrospectively (August 15, 2019; Trial Registration Number: ISRCTN90253431).

## Introduction

Teachers’ emotions are recognized to be of critical importance to the quality and effectiveness of their teaching in the classroom [1–5]. Teachers’ emotional intelligence, assessed in 559 basic and secondary school teachers is positively correlated with their ability to maintain classroom discipline [6]. Teachers’ emotions also influence their health and psychological well-being [7].

Yoga is known to positively influence physical and mental well-being [8]. Previously, the same authors assessed primary school teachers in India (n = 118, both sexes) who, after 15 days of yoga in a residential setting had an increase in mental well-being (small Cohen’s d effect size = 0.36) and a decrease in state anxiety (small Cohen’s d effect size = 0.25), compared to an equal number of their colleagues who carried on with their routine [9].

Professional well-being was evaluated in teachers in relation to individual, professional or organizational factors; data were collected as part of the Teaching and Learning International Survey (TALIS) [10]. Twenty-three countries took part in the survey with participation from 72,190 teachers from 4401 schools. Multiple hierarchical regression analyses were carried out to determine which categories of variables, i.e., individual, professional or organizational could maximally influence teachers’ professional well-being. Individual factors, including gender and level of teaching experience did not powerfully predict professional well-being in teachers.

The present single group study aimed at determining (i) if teachers’ age or gender could predict their baseline levels of mental well-being and anxiety and any change in these variables after yoga. (ii) Whether mental well-being or state anxiety changed following 15 days of yoga in 302 primary school teachers.

## Main text

### Methods

#### Participants

Three hundred and two primary school teachers from an Indian north-eastern state were deputed for a 15 day residential yoga training to a yoga centre in north India. All teachers completed the trial. The state government deputed one teacher per government-funded primary school. The teachers were between 26 and 55 years (group mean age ± SD; 41.83 ± 5.90 years; 192 females). During the yoga training all related expenses were met by the school and the teachers received their full salary.

To be included in the study the participants had to be (i) full time primary school teachers, (ii) naïve to yoga, and (iii) not following any other wellness strategy. The criteria for exclusion included: (i) any illness or chronic use of medication (based on the response to questions) and (ii) the use of alcohol or any intoxicant. None of the teachers were excluded for these reasons. The self-contained, residential campus had a dining hall, a walking track and shops to buy basic necessities.

Participants gave their signed consent to take part in the trial. The trial was approved by the institution’s ethics committee (approval number: PRF/16/0012) and was completed between January 16–January 31, 2016.

With a sample size of 302 and effect size of 0.421 (for mental well-being scores), the power was 1.0000 for alpha pre-set at 0.05, determined using G Power Software [11].

The data of 118 teachers from the 302 teachers presented here were analyzed and published elsewhere by the same authors [9].

#### Design

The study was reported according to standard CONSORT guidelines and was a single group longitudinal study [trial registration number: ISRCTN90253431]. The absence of a control group is a limitation in interpreting the effects of yoga on mental well-being and state anxiety. Participants were assessed at the beginning and end of their 15 day yoga program.

Their day began at 04:00 h and ended at 21:00 h. During the 15 days yoga was taught as practice and theory. Yoga practice was for 240 min (2 sessions per day, each session lasted 2 h). Theory (120 min per day) was derived from the yoga texts, especially the Patanjali’s Yoga *Sutras* (*Circa* 900 B.C.) [12].

#### Assessments

Participants were assessed at the beginning and end of the 15 day yoga intervention. Two questionnaires were used to assess (i) mental well-being and (ii) state anxiety.

#### Mental well-being

The Warwick-Edinburgh Mental Well-being Scale was used [13]. It has 14 items, with five options: never (scored as 1), rarely (‘2’), sometimes (‘3’), often (‘4’) and very often (‘5’).

#### Spielberger’s State Trait Anxiety Inventory—State (STAI-S)

State anxiety was assessed with a sub-scale of the Spielberger’s State-Trait Anxiety Inventory with 20 items to assess state anxiety (i.e., anxiety at the moment of assessment) [14]. Participants chose the number which most appropriately described the intensity of their feelings at the moment of testing: “1” for “not at all”, “2” for “somewhat”, “3” for “moderately”, and “4” for “very much so”.

#### Yoga Intervention

The yoga intervention was for 15 days with two practice sessions each day. Each session was for 120 min. In practice sessions the participants were taught: (i) loosening exercises, (ii) physical postures (*asanas*), (iii) breathing techniques (*pranayamas*), and (iv) meditation and relaxation. The details are given in Table 1. Also, participants were given practical suggestions to achieve a calm mental state and have healthy coping strategies to manage stressors. This information was in a 2 h theory session every day. The information was derived from yoga texts, such as the Patanjali’s Yoga *Sutras* (*Circa* 900 B.C.) [12].

**Table 1:** Details of a yoga practice session

No.	Type of the practice	Name of the practice	Duration (min)
1	Meditation	On *sanskrit* syllable, OM	10
2	Warming up	Sun salutation (*Surya namaskara*)	10
3	Loosening exercises	i. Neck rotation	20
		ii. Knee and ankle rotation	
		iii. Shoulder rotation	
		iv. Butterfly pose	
4	Postures (*Asanas*)		30
4.1	Standing postures	Swaying palm tree pose (*Tirryaktadasana*)	
		Angle pose (*Konasana*, both sides)	
		Feet and hands pose (*Padahastasana*)	
4.2	Sitting postures	Frog pose (*Mandukasana*)	
		Rabbit pose (*Sasakasana*)	
		Sitting lateral twisting pose (*Vakrasana*, both sides)	
		Cow face pose (*Gomukhasana*, both sides)	
		Mill churning pose (*Chakkiasana*)	
4.3	Prone postures	Crocodile pose (*Makarasana*)	
		Cobra pose (*Bhujangasana*)	
		Half-locust pose (*Salabhasana*)	
4.4	Supine postures	Half-plough pose (*Ardhhalasana*)	
		Rotating leg pose (*Padavritasana*, both sides)	
		Leg circling pose (*Dwichakrasana*, both sides)	
5	Relaxation	Supine relaxed pose (*Shavasana*)	10
6	Yoga breathing series (*pranayamas*)	Yoga bellows type breathing (*Bhastrika*)	20
		High frequency yoga breathing (*Kapalabhati*, 1.0 Hz)	
		Alternate nostril yoga breathing (*Anulom*-*vilom*)	
		Bee breathing practice (*Bhramari*)	
		Victorious breath (*Ujjayi*)	
		Om chanting (*Udgeeth*)	
7	Relaxation	Supine relaxed pose (*Shavasana*) with breath awareness	10
8	Meditation and concluding prayer		10

Total duration of the session was 120 min and the same session was repeated in the evening

Yoga sessions were conducted in a hall where 4000 people can practice yoga simultaneously. The 302 teachers were seated in 20 rows and 16 columns (with 16 participants in each row) separated by 3.0 feet. The yoga sessions were led by an expert in yoga with over 45 years of experience in the practice and theory of yoga. Also, there were 20 yoga teachers with a minimum of 5 years of yoga teaching experience as assistants. Audio-visual aids helped ensure that the yoga expert, who practiced yoga at the front of the hall on a raised stage, was visible and audible to all participants. Instructions were in Hindi, a microphone was used with 24 speakers, each with an output of 136 dB. There were also two LED screens (10 × 16 feet each) at the front of the hall, approximately 200 feet from the last row.

#### Data extraction and analyses

The data were analyzed using SPSS Version 24.0. The group average values ± SD and Cohen’s effect size for the mental well-being scores and scores for state anxiety were calculated.

There were two types of analyses.

i. These analyses determined whether age or gender could predict mental well-being or state anxiety at (A) baseline or Pre-yoga and (B) after 15 days of yoga practice and theory.
ii. Comparison of post data versus pre was with the *t* test for paired data. The t and p values are presented in Table 2.

**Table 2:** Changes in positive and negative emotions after 15 days of yoga intervention

No.	Variables	Yoga intervention		Percentage change (direction of change)	Cohen’s d-value	t-value	P-value
		Before	After				
1	Mental wellbeing scores	52.08 ± 7.35	55.12 ± 7.07***	5.84 (↑)	0.421	− 7.932	< 0.001
2	Anxiety scores	34.12 ± 8.43	32.59 ± 8.36**	4.48 (↓)	0.182	3.149	0.002

Values are group mean ± SD

** P < 0.01; ***P < 0.001 (two tailed) level of significance, comparing pre and post positive (mental wellbeing) and negative (anxiety) emotions using paired t-test

## Results

None of the participants reported any adverse effects. This was specifically checked after the yoga practice sessions.

i. Age and gender as predictors of mental well-being or state anxiety Pre-Yoga and Post-yoga.
  Gender acted as a significant predictor for mental well-being scores at baseline (Pre-Yoga) {adjusted R^2^ = 0.036; beta coefficient = − 0.197; P = 0.001, 95% CI of [− 4.746, − 1.317]; tolerance = 1.000; variance inflation factors (VIF) = 1.000}.
  Gender significantly predicted state anxiety scores at baseline/Pre-Yoga {adjusted R^2^ = 0.023; beta coefficient = 0.162; P = 0.005; 95% CI of [0.885, 4.841]; tolerance = 1.000; variance inflation factors (VIF) = 1.000}.
  Neither gender nor age acted as predictors for the change in mental well-being or state anxiety Post-Yoga (P > 0.05 in both cases).
(ii) Comparison of Post versus Pre data.
  There was a significant increase in mental well-being in the 302 teachers after yoga compared to before (P = 0.002). State anxiety scores were significantly lower after yoga compared to before (P < 0.001). The pre and post values of mental well-being and state anxiety scores are provided in Table 2.

### Discussion and conclusion

Gender acted as a significant predictor for 302 primary school teachers’ mental well-being and state anxiety pre-yoga, with females showing higher anxiety scores and lower mental well-being scores.

Earlier, data were collected as part of the Teaching and Learning International Survey (TALIS) [10]. Twenty-three countries took part in the survey with participation from 72,190 teachers from 4401 schools. Multiple hierarchical regression analyses were carried out to determine which categories of variables, i.e., individual, professional or organizational would most influence the teachers’ professional well-being. Gender and level of teaching experience were not powerful predictors of professional well-being in the teachers of the survey. These findings are in contrast to the present results where gender did significantly predict mental well-being pre-yoga.

The present study assessed general mental well-being using the Warwick-Edinburgh scale. Mental well-being is defined as a state of physical and psychological good health [15]. Teachers’ professional well-being involves more specific factors such as job satisfaction, self-efficacy, aspiration, motivation and authority [10]. The difference between the present results and those reported earlier may be attributed to the focus of the present study being overall mental well-being, whereas the earlier study reported the effects of gender on teachers’ professional well-being [10]. While some earlier studies reported that gender has an effect on professional well-being [16], it appears as if teachers’ professional well-being (TPW) is chiefly influenced by job-related factors such as the school climate, co-operation among teachers, fair and helpful assessments and the relationship between the principal and the teachers, while individual factors such as gender have less impact on TPW. The present study reported that gender predicted differences in (overall) mental well-being.

Gender acted as a predictor for the pre-yoga scores, with females showing higher anxiety scores and lower mental well-being scores. This finding is supported by a study on gender differences in emotional responses, in which a functional magnetic resonance imaging (fMRI) study was carried out on 25 young adults (13 female) to compare the responses of both sexes when participants were asked to cognitively evaluate and down-regulate their responses to negatively valenced pictures [17]. The results suggested that females process emotional experiences more complexly than males, which also included simultaneously appraising situations in both negative and positive ways.

The primary school teachers showed a significant increase in mental well-being and decrease in state anxiety after 15 days of yoga compared to their pre-yoga scores.

The increase in mental well-being and decrease in state anxiety are comparable to results reported elsewhere for a sub-sample of 118 teachers from the present group studied by the same authors [9]. When the earlier (n = 118) [9] and the present study (n = 302) were compared, there were no clear differences in the effects of yoga, as in both studies there was an increase in mental well-being (n = 302, Cohen’s d = 0.421, 5.84% increase for the present study; n = 118, Cohen’s d = 0.361, 4.95% increase for the earlier study) and decrease in state anxiety (n = 302, Cohen’s d = 0.182, 4.48% decrease for the present study; n = 118, Cohen’s d = 0.246, 5.89% decrease for the earlier study).

In the earlier study [9] the yoga group were compared with an equal number of teachers in a control group who continued with their regular teaching schedule. The control group showed no change in mental well-being or state anxiety. This suggests that repeating the tests after 15 days may not cause a change in well-being or anxiety; however this cannot be conclusively stated for the present study where there was no control group for the 302 teachers who practiced yoga.

## Limitations

The limitations include (i) the absence of a control group, (ii) the present yoga program was residential and intensive; it would be ideal to test a yoga program which can be incorporated in the work day and (iii) since the data were not blind scored there could be bias. The results cannot be considered conclusive due to these limitations, but the results suggest that it would be worth evaluating yoga to positively impact teachers’ overall health and performance.

## Data Availability

The individual data are available in the archives of the laboratory and can be obtained from the corresponding author on request.

## Abbreviations

B.C.: before Christ
CI: confidence intervals
CONSORT: consolidated standards of reporting trials
dB: decibels
fMRI: functional magnetic resonance imaging
ISRCTN: international standard registered clinical/soCial sTudy number
LED: light emitting diodes
PRF: Patanjali research foundation
SD: standard deviation
SPSS: statistical package for social sciences
STAI-S: Spielberger’s state trait anxiety inventory-state
TALIS: teaching and learning international survey
TPW: teachers’ professional well-being
VIF: variance inflation factors
